# Mapping HIV-related services for women in Eastern Canada: A qualitative study

**DOI:** 10.1177/17455057221092264

**Published:** 2022-04-17

**Authors:** Priscilla Medeiros

**Affiliations:** Women’s College Research Institute, Women’s College Hospital, Toronto, ON, Canada

**Keywords:** Canada, HIV/AIDS, mapping, qualitative geographical information systems, women

## Abstract

**Background:**

Geographic health disparities have been well described in parts of Canada; however, little is known about the experiences of women living with HIV in the Maritime Provinces. This article focuses on the complex health system women living with HIV navigate geographically to access care in New Brunswick and Nova Scotia, Canada.

**Method:**

This study includes interviews with 10 women living with HIV and 39 community-based workers whose organizations provide services to this group of women in New Brunswick and Nova Scotia. Purposive sampling was used to recruit both women living with HIV and community workers. Interviews were recorded and transcribed into a Microsoft word document. Transcripts were imported into NVivo 11 for thematic analyses and used to map the services women with HIV were accessing in their communities in ArcGIS 10.2 for Windows.

**Results:**

The study found that there are a number of barriers women with HIV face in the Maritime Provinces, including the low number of specialist physicians, long travel distances to major urban centers for care, and the loss of HIV-specific supports and resources. In response to these difficulties, community-based organizations are leading efforts in their communities to increase outreach programs and the number of available peer workers to improve the health outcomes of women living with HIV. Furthermore, it showed that women living with HIV and community workers were interested in creating a women-centered HIV care system in the Maritime Provinces, but were uncertain how to move forward with this initiative.

**Conclusion:**

There is a need for women-centered HIV services. This study proposes streamlining the healthcare pathway and decreasing obstacles to increase women’s access to care in the Maritime Provinces.

## Introduction

The Public Health Agency of Canada estimates 62,050 people are living with HIV in Canada.^1^ The province of Ontario followed by Quebec, Alberta, and British Columbia accounts for the highest number of reported HIV cases in the country.^2^ Women account for less than half of the people living with HIV in Canada. Despite evidence that the absolute number of women living with HIV is increasing over time, they remain an understudied group, particularly in the Maritime Provinces.^1^

The provinces of New Brunswick and Nova Scotia are two of four Atlantic Provinces on the east coast of Canada. According to the latest census figures, the total population in New Brunswick is 776,827 people and in Nova Scotia it is 971,395 people. This is a small population size compared with the rest of the country where Canada has an estimated population of 37.8 million people.^3,4^ Nearly 46% of the total population of New Brunswick and Nova Scotia is rural.^5^ Rural, in this case, refers to “smallness, isolation . . . strong community feelings, [and] conservative and traditional values”^6^ (p. 478). The number of people living with HIV in these regions is unknown due to their low absolute number in these provinces. There is, however, evidence that people with or at risk of contracting HIV who live in rural communities face complex challenges similar to those faced by persons living in urban centers, including poverty, transportation issues, religious conservatism, and a lack of services.^5,7,8^ As it happens, New Brunswick and Nova Scotia are two of the poorest provinces in the country. The government funding they receive is insufficient in meeting the long-term demands of people living with HIV.^6,9^

There are 1832 people with HIV in the provinces of New Brunswick and Nova Scotia. However, New Brunswick has seen an overall 175% increase in HIV incidence and Nova Scotia a 100% increase among the entire population.^10,11^ Women living with HIV represent 13% (*n* = 238) of these cases.^10,12^ Newly reported HIV infections in females are mainly attributable to heterosexual sex and intravenous drug use.^10,13^ Women living with HIV in both provinces are less likely than men to be tested for HIV and to access treatment because of social stigma biased toward men.^14^ These complex situations underpin the reasons why women living with HIV in the Maritime Provinces demand a stronger referral network to better access health and social services in their area.

The geographic location and accessibility of HIV-related services in the Maritime Provinces have received little attention in the literature, despite the availability of women-only HIV services across the country, such as the Oak Tree Clinic in Vancouver, Maple Leaf Medical Clinic in Toronto, Women’s Health in Women’s Hands Health Centre in Toronto, and the Centre for AIDS Services of Montreal.^15,16^ Health geography lends itself well to understanding the relationships between place and health. It focuses on the study of access to healthcare services to understand the geographic locales of disease.^17^ A combination of qualitative interviews and geographic information systems (GIS) mapping techniques has been used to identify areas where there are significant clusters of health services for women living with HIV in the two provinces and gaps in care.

## Methods

### Study design

This is a multi-sited study that combines interview discussions and GIS to map the location of HIV-related services for women with HIV in New Brunswick and Nova Scotia. The use of maps in this study provides a visual record of the location of these services, and to identify gaps in care for women with HIV in the two provinces, individual semi-structured interviews were conducted with 10 women living with HIV and 39 community workers who provide services to this group of women in New Brunswick and Nova Scotia. Participants were asked to share their accounts regarding the overall quality of care experienced by people living with HIV, gaps within primary care, and the geographic location of HIV-related services. Because community-based workers are often the first point of contact for people living with HIV and make referrals to HIV-related services, they were included as participants in the study to map the locations of these services and discuss the gaps in care from their perspective. The experiences and perspectives of both women with HIV and community-based workers were vital to mapping and examining the availability of all of HIV-related services in the two provinces.

This study follows the theoretical approach of critical medical anthropology (CMA) to examine the changing health priorities of women living with HIV as they navigate the healthcare systems after diagnosis. CMA focuses on the individual’s perspective to better understand health issues.^18–20^ It also values the input of community stakeholders at every stage of the research.^21,22^ This study was reviewed and approved by the McMaster University Research Ethics Board.

### Participant recruitment

**Figure 1.**
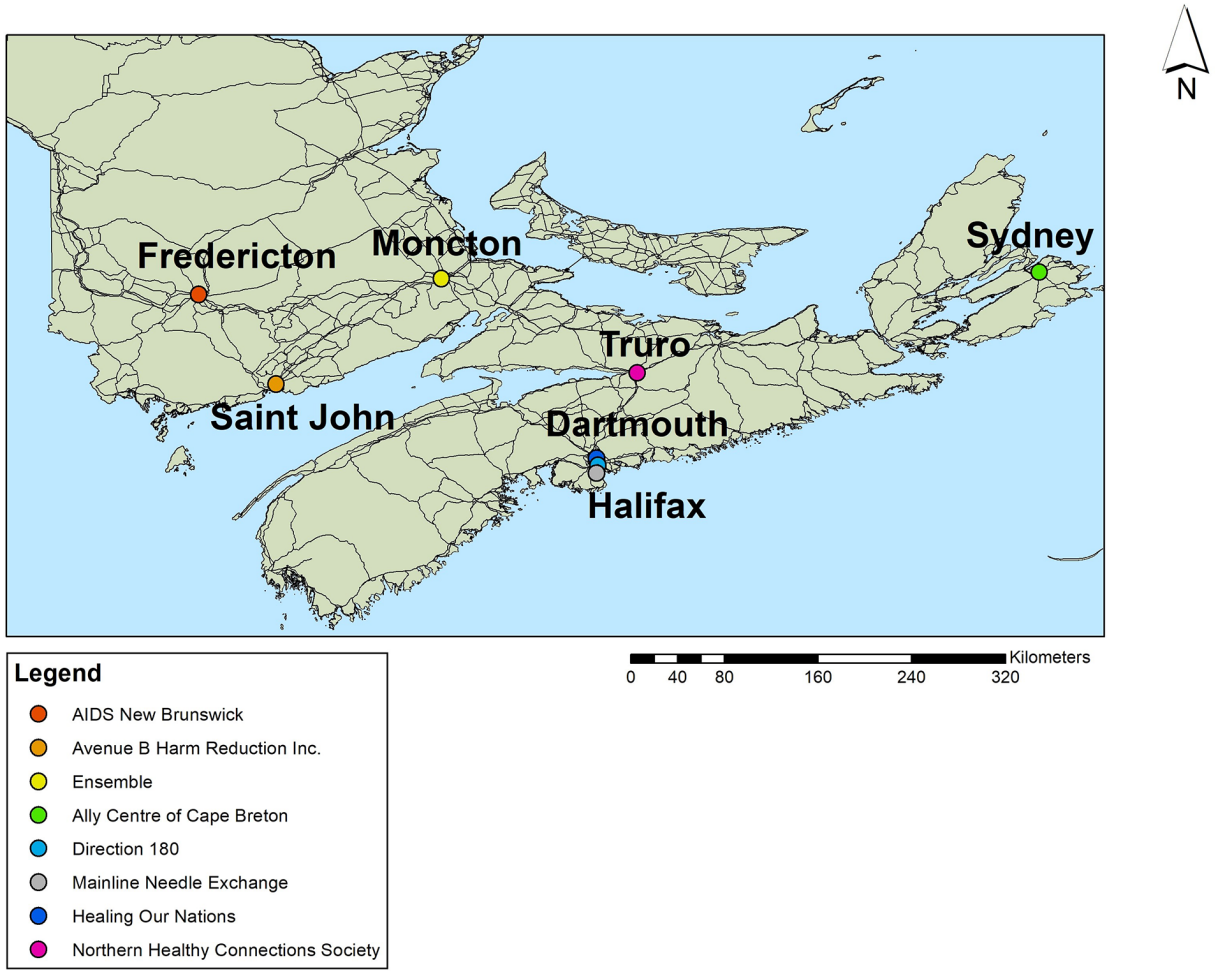
Map of multisite study. Created by Author using ArcGIS 10.2.

The author approached eight community-based organizations (CBOs) in New Brunswick and Nova Scotia to participate in this study. Figure 1 shows a map of where the multi-site project took place in New Brunswick and Nova Scotia.

Eight CBOs were chosen as they are often the first point of contact for women in the Maritime Provinces after they receive an HIV diagnosis. Testing sites refer women to these CBOs for community referrals, and as such, these organizations were important partners in selecting a diverse range of participants and communicating findings with them.

Recruitment flyers were placed in the offices of eight CBOs and word-of-mouth from community workers served as the primary methods for recruitment. The project was also advertised through other communication channels across the two provinces, including medical providers, food banks, and transition housing for women. Community workers also contacted women with HIV who were accessing services from their organization to inform them of the study and ask whether they would be willing to participate. Community workers then helped with screening women living with HIV to determine their eligibility. Requirements included the following: being 18 years of age or older; self-identifying as a woman living with HIV (inclusive of *cis* or *trans* women); living in New Brunswick or Nova Scotia at the time of the study; and willing and able to participate in an audio-recorded interview.

Community workers who serve women living with HIV were also interviewed to develop an understanding, from multiple perspectives, of positive women’s views on barriers to care in both provinces. The eligibility requirements for community workers to participate in this study included the following four criteria: directly interacting with women living with HIV as part of their central mandate; based in New Brunswick or Nova Scotia; willing and able to participate in an audio-recorded interview; and staff at one of the recruiting CBOs.

The author also screened potential community workers via telephone to determine their eligibility to participate and contacted women living with HIV and community workers directly for participation, obtaining verbal consent from participants to record and transcribe their interview.

### Data collection

Interviews were conducted between June 2014 and March 2015 in seven cities in New Brunswick and Nova Scotia. These sites are where most women living with HIV access HIV services in both provinces, including sexual and reproductive healthcare services, mental health services, and HIV care and treatment. A purposive sampling approach was used to include a variety of viewpoints from both women living with HIV and community workers.^23^ Ten women living with HIV and 39 community workers participated in this research.

**Figure 2.**
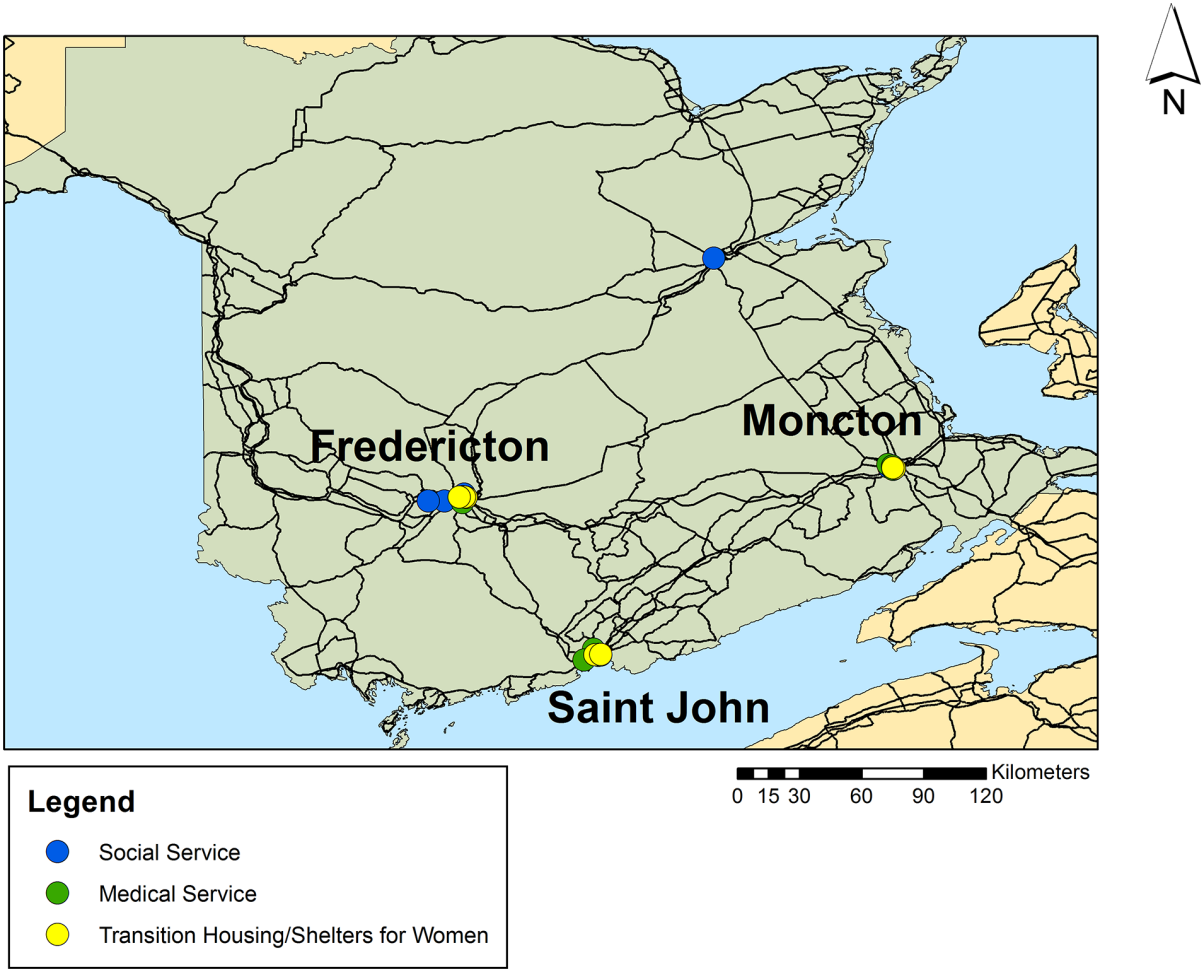
Referral network locations for women living with HIV in New Brunswick. Created by Author using ArcGIS 10.2.

For the interviews, the author developed an in-depth guide after an extensive literature review regarding qualitative GIS and the visualization of narratives. The author’s supervisor and three community-based workers approved the interview guide (see Supplemental Appendices I and II). Each participant took part in a 1–1.5-h long one-on-one interview. Following each interview, a debriefing session was held with participants to discuss ways of improving women’s access to services in the region. All participants read a study information sheet and signed a consent form to participate in this research before data collection commenced. The author also obtained consent for publication from all participants. Participants were also assigned pseudonyms to assure anonymity and to protect participants’ privacy. The interviews were audio-recorded and transcribed verbatim. The geographic locations of access to services for women living with HIV were identified from the audio-recordings. Resource materials located in the offices of CBOs were also helpful in determining what services and supports women were accessing in their community to expand this list.

### Data analysis

Interview transcripts were analyzed and managed using NVivo 11 qualitative data analysis software for Windows. Analysis began with the author independently reading, highlighting, and annotating transcripts to identify the HIV services women access for HIV treatment and care following Creswell’s process of narrative analysis.^24^ Next, a codebook was developed to document and organize codes that included *women’s access* to *services* and *resources.* All transcripts were coded using the final codebook. The author discussed the coding with an advisory committee when discrepancies emerged until a consensus was reached. The narratives that clearly described women’s stories of access to care challenges in New Brunswick and Nova Scotia formed the basis of this study.

As part of this analysis, the author used participants’ responses to healthcare access in their communities to map out the most common HIV services and resources women with HIV access in New Brunswick and Nova Scotia. All map-based spatial analyses were completed using ArcGIS 10.2 for Windows.

## Results

Maps were created using ArcGIS 10.2 that visually highlight the locations of HIV-related services for women in New Brunswick and Nova Scotia. It also demonstrates the increased dependence women living with HIV have on CBOs to navigate services to meet their basic food, housing, and transportation needs.

### Participants

There were 10 women living with HIV involved in this study. They ranged in age from 38 to 75 years (median, 46 years of age), and on average had been living with HIV for 14 years. All of the women were in good health at the time of the study. All of the women also self-identified as Caucasian despite the diverse screening criteria used to reach racial and ethnic populations, which is representative of the people living with HIV in both provinces. Four of the women were single at the time of the interview, whereas six were widowed, divorced, and/or married. In addition, nine of the women lived in an urban setting. The decision to live or move to an urban area was brought on by the desire to improve access to care. Regardless of where women lived, perhaps the most striking finding was that six women were relying on social assistance after diagnosis because of their illness and the difficulties of entering the labor market at older ages. The women had a median annual income of CAD10,800 ranging from CAD6,000 to CAD30,000. Only one woman reported earning more than CAD30,000 per year. These reported annual incomes remain relatively the same to-date.

Thirty-nine community workers at all levels of participating CBOs were interviewed in this study. Community workers ranged in age from 25 to 59 years of age (median, 43 years of age), and 10 out of 39 were also living with HIV, hepatitis C, or both viruses. Of the community workers enrolled in this study, 85% self-identified as Caucasian, 2% as Black Canadian, and 13% as Indigenous. In addition, most of the community workers were women (69%) and held leadership positions in the organization including executive director and community outreach specialist.

### HIV-related services for women

All of the CBOs involved in this study work closely with other non-profit organizations, local doctors and nurses, and community members to provide women with care and support to meet their unique health needs after a positive HIV diagnosis.

**Figure 3.**
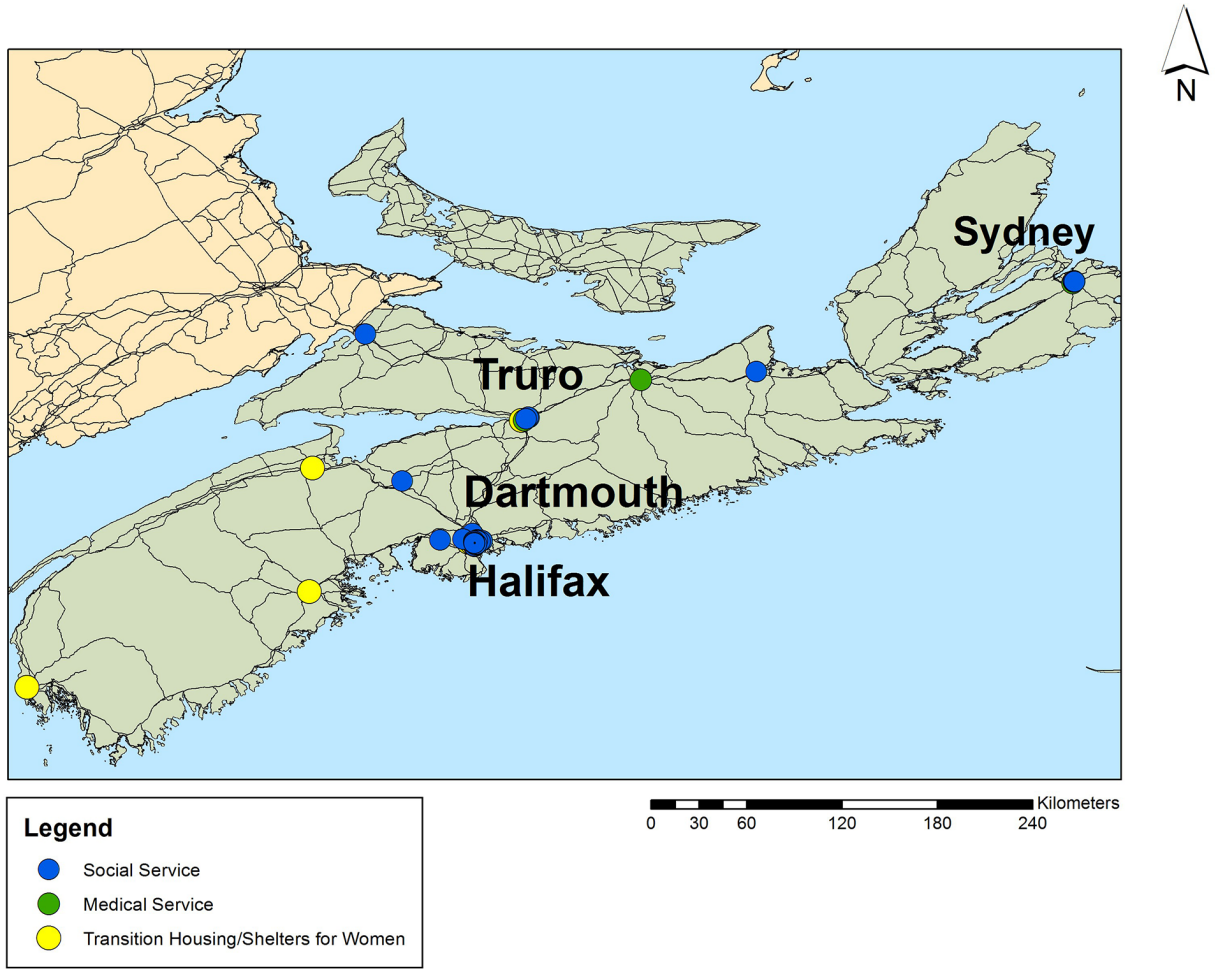
Referral network locations for women with HIV in Nova Scotia. Created by Author using ArcGIS 10.2.

Figures 2 and 3 show how referral network locations are clustered within the urban locations of New Brunswick and Nova Scotia. Both maps reveal a duplication of services through the clustering of points, which adds further complexity to navigating the care system in these provinces, particularly from the perspective of community workers who serve them in making the referrals to local supports.

**Figure 4.**
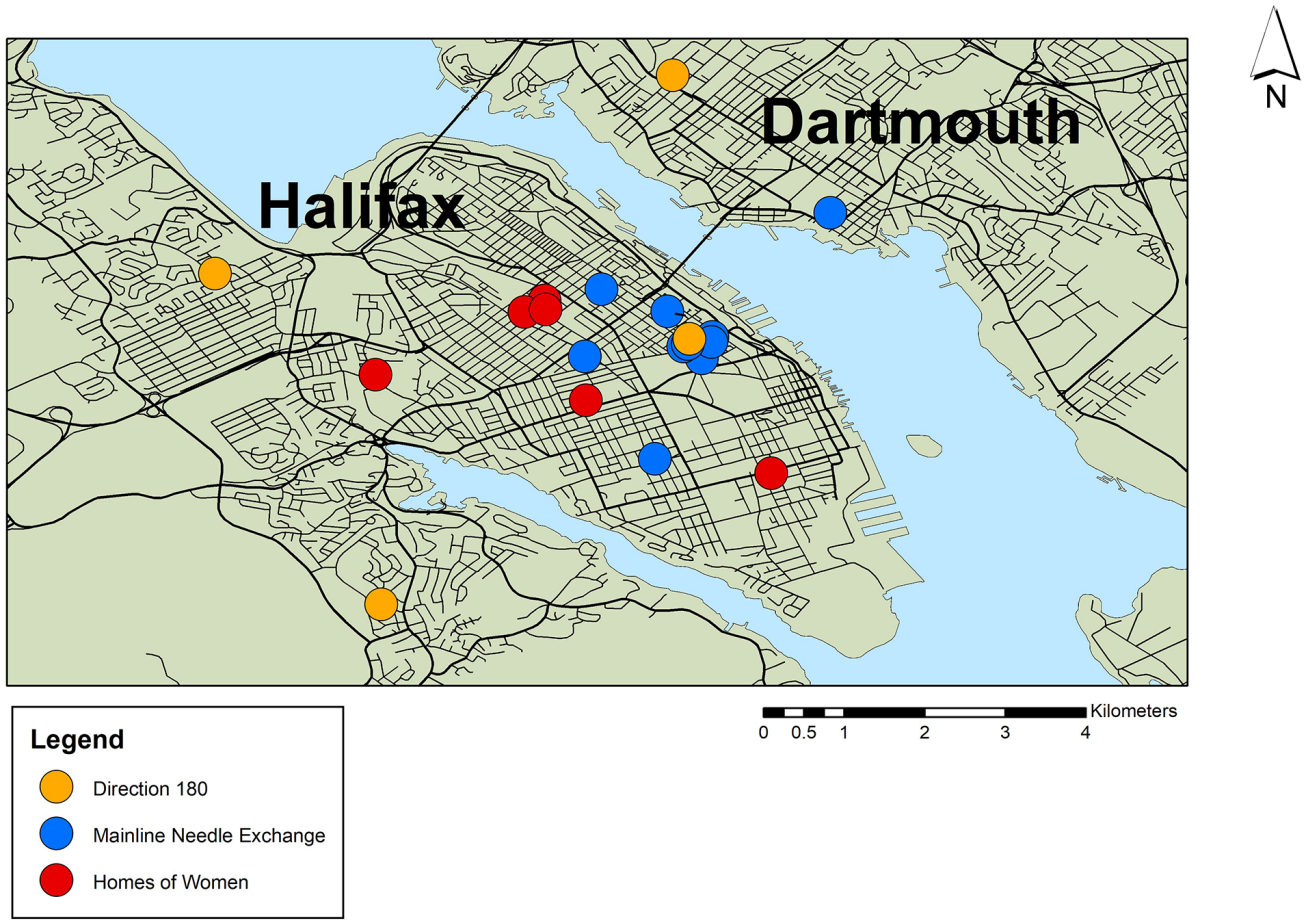
Mobile care sites in Halifax and Dartmouth, Nova Scotia, to support people living with HIV and hepatitis C. Created by Author using ArcGIS 10.2.

Despite the many available HIV services in New Brunswick and Nova Scotia, only 15% of the existing referrals for women living with HIV displayed in Figure 4 are found in rural areas of the province. Most services women are accessing run along fixed routes of the public transit system in urban areas, which are often less than a 5-km distance from each other. In some cases, women would walk short distances to their appointments to access services.

To help women with HIV who have limited access to local community resources and services, local community organizations (e.g. Mainline Needle Exchange, Mobile Outreach Street Health, and Direction 180) have deployed a mobile medical unit in the Halifax Regional Municipality. These community organizations provide treatment, prevention, and recovery services to people living with HIV and other sexually transmitted and blood-borne infections. Figure 4 shows the location of daily mobile outreach services in Halifax and Dartmouth. The mobile unit delivers onsite care and community supports, including harm reduction supplies (e.g. condoms, swabs, cookers, Vitamin C), wound care, addiction-related support, health education, and community referral support.

Mobile outreach units often provide an entry point for and at times directly provide HIV services for women. One community worker from Direction 180 went on to explain how the mobile methadone program provides outreach to underserved populations:

We have four sites located all throughout the metropolitan area that we visit. The mobile unit was started [when the] metro transit had gone on strike a few years back. . .The amount of people actually coming and accessing the treatment was amazing [and the organization] got the idea to launch a mobile unit to broaden access and increase adherence to methadone treatment . . . having the mobile unit in communities of need has been important to adherence. (Community Worker, Nova Scotia)

The availability of a mobile unit in the Halifax Regional Municipality enables women with HIV to receive care free of charge that would otherwise have no access to transportation. Although both provinces have established a strong referral network, their capacity to support the long-term needs of women living with HIV remains in question.

### The loss of HIV support programs

All of the women living with HIV involved in this study lacked a support group in their community within which they could discuss health problems unique to women, such as abnormal cervical cytology and bacterial vaginitis, disease progression and changes in the female body, and women’s health needs and human rights. These are common issues women face and want to discuss with others who live with this disease. Two women from Nova Scotia describe the challenges they face in meeting other women living with HIV in their communities:

I’ve only met a few [other] women through the Ally Centre of Cape Breton, which had women’s meetings [in the past] for us to get together, but generally only two or three of us show up. . .those happen a few times a year, but they have been cancelled. . .I think it’s kind of a shame that women don’t get together because I’d like to meet some of the other women with HIV and talk to them. (Woman living with HIV, Nova Scotia)

There are really no groups that you can join to meet other HIV women and speak freely [about women’s health issues]. That’s why I would like to start a group just for women [and] have people living with HIV join [to talk about] how they cope with it and how they live with it. (Woman living with HIV, Nova Scotia)

These comments highlight the need for women to improve their health status by knowing and sharing their experience with other women living with HIV. A low prevalence of women living with HIV in New Brunswick and Nova Scotia, and the loss of HIV support services are main challenges for women to participate in

HIV support groups and general meetings are organized by the community organizations.

### Importance of CBOs in the health system

CBOs are often the first point of contact for women living with HIV after a positive diagnosis. Given organizations’ wider responses to HIV, hepatitis C, and sexually transmitted and blood-borne infections, the ways in which CBOs support people living with HIV have changed:

I think that the services we offer are probably ones that are more advocacy-based . . . helping people to see that there’s a way out of their problem[s]. Let’s sit down and talk about it. What are the options. . .The organization is not funded for support work [by the federal government]. There’s no money for support work. It’s all fundraised. (Community Worker, New Brunswick)

Conversations with long-term community workers point to the growing dependence on government funds to provide education and support services to people living with HIV. The annual Walk for Life, for example, is the main charitable event all CBOs host each year to fund programs to help people living with HIV that might otherwise be missing in communities. The Sex Workers Women’s Drop-in program made available through Avenue B Harm Reduction Inc., formerly known as AIDS Saint John, provides a weekly harm reduction space for women who engage in sex work and may struggle with drug use. This is the only peer-based outreach service for sex workers of its kind in the two provinces for women living with HIV.

Comments from community workers also draw awareness to the importance of community workers as effective counselors for many people living with HIV. Women’s reliance on CBOs to meet their basic needs and improve their quality of life has created a level of trust between community workers and clients that is difficult to replicate with a registered therapist. Trust is the main reason why community workers come to know their clients very well. The following comment from one community worker is a case in point.

What we provide here is one-on-one [support] as needed. They may come by and need someone to talk to. Sometimes they may be having landlord issues and they might need help finding community resources . . . Clients talk about wanting to have social supports, but we have such a small population [of people living with HIV], which is hugely diverse . . . as an agency when we’re told [by the federal government] that we must involve and do programming [to support people living with HIV], how do I that? (Community Worker, New Brunswick)

Community workers are well aware of the support services that women require to meet their unique needs (i.e. food, shelter, and clothing) and continue to work toward addressing these gaps in their communities by creating social services in their organizations through different fundraising activities. The following comments demonstrate the growing challenges CBOs face in making social services available onsite:

It makes sense that what we offer [support services on-site to] help the clients that we serve. If I were a person living with HIV and were to call to ask what they can do for me, I [would] say well we can offer you internet access, we can help with cab fare, we can give information, [and] we can do referrals. The real challenge of meeting their needs is the fact that their needs are very wide and can be very different from one person to the next. We struggle to support peoples changing needs. (Community Worker, New Brunswick)

CBOs depend on engaged community partnerships to meet the long-term health needs of people living with HIV in the face of absent funding to provide their own onsite community resources and support services. They rely on a strong referral network in the face of limiting financial assistance from the government for support work.

## Discussion

CBOs have found creative ways to improve their capacity in building healthy communities, including expanding services for women through existing referral networks, increasing outreach efforts with the help of peer workers, and defining a women-centered approach as envisioned by women living with HIV.

### Increasing women’s health services

The CBOs involved in this study find it difficult to garner sufficient funding for their work and depend on referral services and sharing services with other community organizations to help women living with HIV access necessary supports including treatment, education outreach, and housing and addiction services given how limiting HIV-related services are in the Maritime Provinces. CBOs in response are working together across provincial borders to make sure there is no duplication of efforts.^25^ This, however, is *not unique to New Brunswick and Nova Scotia*. Many community organizations in other rural, small population centers in Canada and elsewhere address the needs of the community (i.e. access to acceptable housing, nutrition, and treatment) through collaboration among health agencies to improve the health of clients.^26^ Very little has been written on the dependency these community organizations have on one another to improve access to care for their clients. Even in the face of extremely limited funding for CBOs, increasing numbers of new HIV diagnoses, and desire for women-only health and support services in this study, collaborating across interprovincial borders is a way to meet the needs of women living with HIV. This type of collaboration continues to happen in New Brunswick, Nova Scotia, and elsewhere and helps organizations break away from knowledge silos.

### Increasing community outreach and peer workers

Mobile outreach programs are important in the comprehensive care of women living with HIV. Mainline Needle Exchange in collaboration with the Mobile Outreach Street Health and Direction 180 have launched two mobile units for those in rural parts of Nova Scotia who can benefit from wound care, addiction-related support, counseling, testing, education, and community referral support. This is a one-of-a-kind treatment service in Mainland Nova Scotia that is distinguished from other mobile outreach services elsewhere in Canada. The mobile unit delivers onsite care and community supports across the Halifax Regional Municipality to ensure the continuity of care for individuals. Similar mobile health clinics exist elsewhere in the world to deliver HIV testing and other basic health services to reach underserved groups.^27–29^ More recently, AIDS New Brunswick launched a new mobile harm reduction unit in Fredericton and surrounding areas to address the increased need for needles and inhalation supplies resulting from the COVID-19 pandemic.^30^ Little else is known about the service given it was launched the summer of 2020.

Peer workers are essential to improving program delivery and health of women living with HIV in communities and provide a liaison between community members accessing the mobile outreach program. Peer workers are important partners in helping women access HIV-related services in New Brunswick and Nova Scotia. They use their lived experience and knowledge, and are trained and supervised by CBOs to provide direct *peer* support and information to clients.^31,32^ Peer support is an important contribution to HIV care and plays an essential role to reduce stigma and improve the health outcomes of others living with HIV.^33,34^ CBO workers, on the other hand, would benefit from additional training on health education and community referral support systems to help the women they counsel.^35^ Avenue B Harm Reduction Inc. (formerly known as AIDS Saint John) based in New Brunswick is the only CBO to rely on peer workers to deliver province-wide outreach activities including harm reduction initiatives, and social and emotional peer support in their own communities. Budget issues often makes it difficult for organizations to pay their peer workers a salary, meaning that many of these peer supports must volunteer their time. Dedicated budgets and staff would increase available peer supports.

### Desire for women-centered HIV care

Women living with HIV want a women-centered care model that includes treatment, education outreach, and housing and addiction services to increase their visibility in the epidemic.^36^ Women-centered approaches to care are largely absent in New Brunswick, Nova Scotia, and other smaller provinces, including Manitoba and Prince Edward Island, because of organizational funding challenges.

A number of recent studies on women-centered HIV care in Canada demonstrate the importance of gendered approaches and their implications for women’s health.^37–39^ These studies show how gendered approaches to care are not only merely related to reproduction and sexual health, but also acknowledge that women’s experiences living with HIV differ from those of men.^39^ All of the participants in this study want a space for women to share information and resources with referrals to care, access food programs to meet their dietary needs, and transit tickets to travel to and from appointments, and to talk about living with HIV and wider sexual and reproductive health and rights issues. The delivery of women-centered HIV care elsewhere in the world encompasses the meaningful participation of women in matters regarding their own health, person-centered approaches to care, trauma-informed and safe space practices, healthcare provider training, and promotes women’s care self-management.^40–42^ Women-centered approaches to care are under-researched and underutilized in meeting the needs of women living with HIV. The latest resource from the Canadian HIV Women’s Sexual and Reproductive Health Cohort Study (CHIWOS; www.chiwos.ca) is a comprehensive Women-Centred HIV Care toolkit intended to create integrated services including mental health, sexual and reproductive health services, trauma-informed and safe space practices, healthcare provider training, and women’s care self-management.^43^ All of the interviews with women and community workers revealed feelings of optimism for integrating the toolkit in their communities and increasing more person-centered supports for women. This optimism may be difficult to reconcile with the shrinking budgets of organizations. Therefore, the healthcare landscape in New Brunswick and Nova Scotia also remains relatively unchanged over the past few years.

This study was not without limitations. Establishing trust with women in the community was a main challenge to overcome in this study. Despite time and effort spent building relationships with CBOs, some women declined to participate because of health problems and distrust of the author’s intentions. It was also clear from the outset that the small reported numbers of women living with HIV in the two provinces would pose a challenge to achieve data saturation in a qualitative study. However, studies continue to demonstrate that women’s enrollment and retention in HIV research is unacceptably low.^44,45^ The sensitive nature of the research topic, and presence and experience of stigma, continues to influence women’s willingness to participate in health research; concepts of stigma need to be addressed through different research efforts to increase their enrollment in similar studies.^43^ Despite the strategies used, there remains a risk of bias.

## Conclusion

Generating source maps that combine traditional GIS spatial analyses with qualitative interviews offers an alternative to statistical formats for presenting health information. The GIS maps presented here provides important information for expanding outreach services in the two provinces. In so doing, the maps contribute to making women more visible in the epidemic, especially as spatial data become more widely available to help with decision making. The results of this study are consistent with the previous work of scholars.^15,46–47^ It demonstrates a need for policy-makers and care providers to acknowledge the existing spatial disadvantage for women to access healthcare services and create a landscape of care that better reflects women’s needs and long-term health goals.

Change can be achieved by addressing the essential services that are missing in their communities to improve the quality of life among women with HIV. Important differences exist between the provinces in terms of access to health and social services. However, marked similarities also emerge to reflect the challenges facing women with HIV and community-based organizations to improve community health outcomes. This study has found that gendered approaches to care in Eastern Canada are insufficient and there is a need for further research to improve women’s health in the Maritime Provinces.

## Supplemental Material

sj-doc-1-whe-10.1177_17455057221092264 – Supplemental material for Mapping HIV-related services for women in Eastern Canada: A qualitative study Click here for additional data file. Supplemental material, sj-doc-1-whe-10.1177_17455057221092264 for Mapping HIV-related services for women in Eastern Canada: A qualitative study by Priscilla Medeiros in Women’s Health

sj-doc-2-whe-10.1177_17455057221092264 – Supplemental material for Mapping HIV-related services for women in Eastern Canada: A qualitative study Click here for additional data file. Supplemental material, sj-doc-2-whe-10.1177_17455057221092264 for Mapping HIV-related services for women in Eastern Canada: A qualitative study by Priscilla Medeiros in Women’s Health
