# Supplementary material for: Mapping HIV-related services for women in Eastern Canada: A qualitative study
Source: Womens Health (Lond). 2022 Apr 17;18:17455057221092264. doi: 10.1177/17455057221092264 (PMC9019332; doi:10.1177/17455057221092264)
Supplement: sj-doc-1-whe-10.1177_17455057221092264 – Supplemental material for Mapping HIV-related services for women in Eastern Canada: A qualitative study [file sj-doc-1-whe-10.1177_17455057221092264.doc]

**APPENDIX I**

**INTERVIEW GUIDE FOR COMMUNITY WORKERS**

1. How many years have you been involved in this work? Please tell me about your role with the organization.
2. What are the broad categories of clients that your organization serves?
3. Approximately how many women are registered with your organization?
4. What services and programs are currently available for female clients in your organization?
5. What referral services are your clients accessing in your region?
6. What do you feel are some of the gaps in programming and services for your clients?
7. What are the current strengths and weaknesses of this referral system?
8. Are there any programs or services you would like to be able to offer and cannot?
9. In your opinion, what are the key issues facing women living with HIV in your community? Probe: How do your current services and programs meet these needs?
10. In your opinion, what are the key issues facing AIDS Service Organizations from offering services that are more appropriate for women living with HIV?
11. What resources or changes would need to be made to address these gaps in service delivery?
12. What are some of the changes in service delivery or program funding you have seen or assisted to implement since working for a community-based HIV agency?
13. In your opinion, what are the reasons for changes to service delivery? Provide an example.
14. What are your thoughts and/or feelings about implementing client-centered services?
15. How will the integration of client-centered services for sexually transmitted infections affect service delivery at AIDS Service Organizations?
16. How does the absence or presence of a HIV/AIDS preventative strategy affect service and program delivery?
17. What is the future direction of services and programs for your organization during the program transition period? Please provide an example of a new initiative that will be available at your organization.
