# Supplementary material for: Mapping HIV-related services for women in Eastern Canada: A qualitative study
Source: Womens Health (Lond). 2022 Apr 17;18:17455057221092264. doi: 10.1177/17455057221092264 (PMC9019332; doi:10.1177/17455057221092264)
Supplement: sj-doc-2-whe-10.1177_17455057221092264 – Supplemental material for Mapping HIV-related services for women in Eastern Canada: A qualitative study [file sj-doc-2-whe-10.1177_17455057221092264.doc]

**APPENDIX II**

**INTERVIEW GUIDE FOR WOMEN LIVING WITH HIV**

1. Tell me about your experience of living with HIV and accessing services or programs offered by [*insert name of AIDS Services Organization*].
2. How many years have you been accessing services or programs related to HIV?
3. What other services or programs are you accessing in your community?
4. How often do you access these services or programs?
5. If you do not access services, please explain why.
6. How did you learn about these services or programs?
7. How far do you travel to access these services? Please identify the mode of transportation you are using to access these services.
8. What do you feel are some of the gaps in programming and services for women living with HIV in [*insert name of province*]?
9. How have the services you access changed in the past few years? Please provide me with an example.
10. What are the key issues facing women living with HIV in your community? How do the current services and programs available by AIDS Service Organizations or other service providers meet these needs?
11. In your opinion, have there been any improvements in service delivery for women living with HIV in your community? Please provide me with an example.
12. How would you improve the accessibility of services or programs for women living with HIV?
13. What are the key issues facing AIDS Service Organizations from offering services that are more appropriate for women living with HIV?
14. What are your thoughts and/or feelings about accessing client-centered services at [*insert name of AIDS Service Organization*]?
15. How will the implementation of client-centered services affect your experience as a female client?
16. Do you have any further comments or suggestions on how programs and services can be improved for women living with HIV?
